# Are all cyclin-dependent kinases 4/6 inhibitors created equal?

**DOI:** 10.1038/s41523-019-0121-y

**Published:** 2019-08-29

**Authors:** Antonio Marra, Giuseppe Curigliano

**Affiliations:** 1 Division of Early Drug Development for Innovative Therapies, IEO, European Institute of Oncology IRCCS, Milan, Italy; 2 Department of Oncology and Hematology, University of Milan, Milan, Italy

**Keywords:** Medical research, Cancer, Medical research, Cancer, Medical research

## Abstract

The harnessing in clinical practice of cyclin-dependent kinases 4/6 inhibitors, namely palbociclib, ribociclib, and abemaciclib, has substantially changed the therapeutic approach for hormone receptor-positive metastatic breast cancer (BC). Phase II–III clinical trials evaluating the addition of these agents to standard endocrine therapy reported consistent improvements in response rates and progression-free survival as well as manageable toxicity profiles and excellent impact on patients’ quality of life. Hence, pivotal trials provided comparable results among different cyclin-dependent kinases 4/6 inhibitors, there is an increasing interest in finding substantial differences in order to implement their use in clinical practice. The aim of this paper is to summarize the current evidences raised from preclinical and clinical studies on cyclin-dependent kinases 4/6 inhibitors in BC, focusing on differences in terms of pharmacological properties, toxicity profile, and patients’ quality of life.

## Introduction

Cell cycle dysregulation promotes aberrant cell proliferation and is one of the widely recognized hallmark of cancer.^1^ In this context, the action of cyclin-dependent kinases 4/6 (CDK4/6) is necessary for the transition from G1-to-S phase, being crucial for normal and cancer cell proliferation.^2^ The molecular mechanism underlying these functions includes the activation by D-type cyclin proteins leading to phosphorylation of retinoblastoma-associated protein and E2F protein-mediated transcription of cell cycle genes, such as cyclins A and E.^3^ Given that the CDK4/6-RB1 axis is critical to cell cycle progression, it is to be expected that several tumors disrupt these fine interactions to promote cancer growth.

Hormone receptor (HR)-positive and human epidermal growth factor receptor 2 (HER2)-negative breast cancer (BC) presents different degrees that makes itself susceptible for CDK4/6 inhibition. Cyclin D1 is highly expressed in estrogen receptor (ER)-positive BC, with or without concomitant amplification of the cyclin D1 gene (CCND1). In addition, ER signaling pathway is able to activate CCND1 gene promoter.^4,5^ Cyclin D1 can also stimulate ER transcriptional activity in a CDK 4-independent manner.^6^ On the other hand, cyclin E expression is reported as low in ER-positive BC^7^ and RB mutations are rarely found,^8^ reflecting the dependence of ER-positive BC cells on cyclin D1 to start G1-to-S phase transition. Furthermore, cyclin D1 and CDK 4 are able to guide cell proliferation also in an ER-independent manner.^9^ Given these data, pharmacological inhibition of CDK 4/6 represents an appealing and interesting therapeutic strategy to treat HR-positive BC. Selective CDK 4/6 inhibitors (CDK4/6-Is) have been developed and tested in HR-positive BC patients, mainly in combination with endocrine therapy. To date, three CDK4/6-Is have been evaluated in clinical trials with published results: palbociclib (PD0332991; Ibrance, Pfizer, United States), ribociclib (LEE011; Kisquali, Novartis, Switzerland), and abemaciclib (LY2835219, Verzenio, Lilly, United States). On the basis of the results obtained in pivotal trials, these CDK4/6-Is are United Stated Food and Drug Administration (FDA) and European Medicines Agency (EMA) approved for the treatment of HR-positive metastatic BC (mBC) in combination with aromatase inhibitors or fulvestrant both in first and advanced lines of therapy.

Given that these agents have been developed almost at the same time and comparable results have been provided, there is an increasing interest in finding differences between the three drugs to facilitate their use in clinical practice. In the current paper, we will describe the current evidences raised from preclinical and clinical studies on CDK4/6-Is in mBC, focusing on differences in terms of pharmacological properties, toxicity profile, and patients’ quality of life.

## CDK 4/6 inhibitors in metastatic ER-positive BC care

**Fig. 1:**
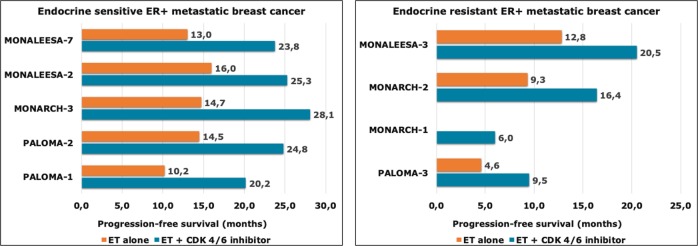
Progression-free survival (PFS) of CDK4/6 inhibitors in clinical trials. Upper and lower panels are referred to endocrine-sensitive and endocrine-resistant settings, respectively

CDK4/6 inhibitors highlighted preclinical and clinical activity in BC, mainly in HR-positive tumors and in combination with endocrine therapy (Table 1). Hence, endocrine therapy is an effective and well-tolerated therapeutic option for patients with HR-positive BC, almost all patients will invariably develop resistance and experience locoregional and/or distant relapses.^10^ A key point to keep in mind when we consider a trial conducted in the metastatic setting of BC is represented by the presence or absence of endocrine resistance to previous treatments. In this regard, the ESO–ESMO International Consensus for Advanced Breast Cancer provides accurate definitions, by distinguishing between primary and secondary (acquired) endocrine resistances.^11^ Pivotal trials that tested CDK4/6-Is in mBC have been conducted both within endocrine-sensitive and endocrine-resistant setting. Figure 1 schematically summarizes the survival gains obtained in clinical trials that are described below.

**Table 1:** Completed phase II–III clinical trials investigating CDK4/6 inhibitors in hormone receptor-positive metastatic breast cancer (mBC)

Trial	Study design	Randomized	Phase	Sample size	Population	Experimental arm	Control arm	PFS (months)	OS (months)
Paloma-1	Open label	Yes 1:1	2	165	AI sensitive Treatment naive for mBC Postmenopausal	Palbociclib plus Letrozole	Letrozole	20.2 vs 10.2 HR 0.48	37.5 vs 34.5 HR 0.89 (NS)
Paloma-2	Placebo control	Yes 2:1	3	666	AI sensitive Treatment naive for mBC Postmenopausal	Palbociclib plus Letrozole	Letrozole plus placebo	24.8 vs 14.5 HR 0.58	NA
Paloma-3	Placebo control	Yes 2:1	3	521	Endocrine resistant Pre/peri and postmenopausal	Palbociclib plus Fulvestrant	Fulvestrant plus placebo	9.5 vs 4.6 HR 0.46	34.9 vs 28.0 HR 0.81
Monarch-1	Open label	No	2	184	AI resistant CT treated mBC Postmenopausal	Abemaciclib	///	6.0	22.3
Monarch-2	Placebo control	Yes 2:1	3	669	AI resistant CT naive for mBC Pre/peri and Postmenopausal	Abemaciclib plus Fulvestrant	Fulvestrant plus placebo	16.4 vs 9.3 HR 0.55	NA
Monarch-3	Placebo control	Yes 2:1	3	493	Endocrine sensitive Postmenopausal	Abemaciclib plus Anastrozole/Letrozole	Anastrozole/Letrozole plus placebo	28.1 vs 14.7 HR 0.54	NA
Monaleesa-2	Placebo control	Yes 1:1	3	668	Endocrine sensitive Treatment naive for mBC Postmenopausal	Ribociclib plus Letrozole	Letrozole plus placebo	25.3 vs 16.0 HR 0.56	NA
Monaleesa-3	Placebo control	Yes 2:1	3	725	Endocrine sensitive and endocrine resistant mBC Postmenopausal	Ribociclib plus Fulvestrant	Fulvestrant plus placebo	20.5 vs 12.8 HR 0.59	NA
Monaleesa-7	Placebo control	Yes 2:1	3	672	Endocrine sensitive Pre/perimenopausal	Ribociclib plus Anastrozole/Letrozole plus LH–RH analog	Anastrozole/Letrozole plus LH–RH analog plus placebo	23.8 vs 13.0 HR 0.55	NR vs 40.9 HR 0.71

*HR* hazard ratio, *LH–RH* luteinizing hormone-releasing hormone, *NA* not available, *NR* not reached, *NS* not significant, *OS* overall survival, *PFS* progression-free survival

## Palbociclib: PALOMA trials

Palbociclib is a reversible, small-molecule CDK4/6-I and was the first-in class to show meaningful clinical activity in ER-positive BC both in preclinical and clinical studies.^12,13^ Palbociclib showed a highly specific activity against CDK4 and CDK6, with a half maximal inhibitory concentration (IC_50_) of 11 and 15 nanomol/Litre (nM/L), respectively.^14^ In addition, it demonstrated antiproliferative activity against RB-positive tumor cells in vitro, inducing an exclusive G1 arrest with a concomitant reduction of phospho-Ser780/Ser795 on the Rb protein.

In the Phase II open-label randomized PALOMA-1/TRIO-18 trial, palbociclib plus letrozole significantly increased progression-free survival (PFS) as compared with letrozole alone in treatment-naive, postmenopausal HR+/HER2− mBC patients (20.2 versus 10.2 months).^15^ Of note, the cohort 2 of PALOMA-1/TRIO-18 trial enrolled patients with defined molecular alterations, such as CCND1 amplification, loss of p16 or both. However, no predictive role of these biomarkers on palbociclib efficacy was observed. In elderly patients (age > 65 years), median PFS confirmed to be significantly augmented in palbociclib arm compared with letrozole-alone arm (26.2 vs 12.9 months).^16^ An updated analysis revealed that palbociclib plus letrozole did not increase median overall survival (OS) compared with letrozole alone (37.5 versus 34.5 months, HR 0.89, *p* = 0.28).^17^ Based on the encouraging results of this trial, a Phase III randomized study was conducted. The Phase III randomized clinical trial PALOMA-2 enrolled patients with treatment-naive, endocrine-sensitive postmenopausal HR+/HER2− mBC. Palbociclib plus letrozole significantly improved median PFS compared with letrozole plus placebo (24.8 vs 14.5 months).^18^ PSF benefit was maintained in elderly population. To date, OS data are still immature.

Furthermore, palbociclib has been also tested in endrocrine-resistant HR+/HER2− mBC with the Phase III PALOMA-3 trial that compared the combination of palbociclib and fulvestrant to fulvestrant and placebo in pre- and postmenopausal HR+/HER2− mBC patients whose disease either relapsed during or within 1 year after the completion of adjuvant endocrine therapy or progressed in prior hormonal treatment for metastatic disease. Although the combination of palbociclib to fulvestrant showed an increased median PFS (9.5 vs 4.6 months) as compared with fulvestrant monotherapy,^19,20^ the updated results of this trial did not demonstrate a statistically significant OS improvement for the combination (median OS 34.9 vs 28.0; hazard ratio, 0.81; *P* = 0.09).^21^ Interestingly, the addition of palbociclib to fulvestrant in patients with sensitivity to previous endocrine therapy, either in the context of metastatic disease or in the adjuvant setting before recurrence, highlighted OS and PFS gains of 10 and 7.8 months, respectively. Conversely, these survival benefits were not achieved in patients with intrinsic endocrine resistance.^21^

Unlike PALOMA-1 and PALOMA-2, PALOMA-3 study was the first Phase III trial testing a CDK4/6-I to enroll also premenopausal patients. Among 108 premenopausal HR + mBC patients, median PFS was 9.5 in the palbociclib arm (palbociclib plus fulvestran and goserelin) versus 5.6 months in placebo arm.^22^ Regarding elderly population, 129 patients aged > 65 were enrolled. Survival benefit of older patients was comparable to that of younger population. Interestingly, Turner et al.^23^ recently published a biomarker analysis from mBC patients enrolled in the PALOMA-3 trial. The authors reported that high cyclin E1 (CCNE1) mRNA expression was associated with a reduced PFS in patients treated with palbociclib when compared with those with low expression. These findings were also confirmed in a validation cohort of BC patients from the Preoperative Palbociclib study, suggesting a potential role for CCNE1 as predictor of resistance to CDK4/6 inhibition.

## Abemaciclib: MONARCH trials

Abemaciclib is also a highly selective, reversible CDK 4/6 inhibitor. Among all three, CDK 4/6-Is available in clinical practice, abemaciclib is the most potent with reported IC50s of 2 nm and 10 nm for CDK4 and CDK6, respectively.^24^ In addition, abemaciclib has shown a potential for crossing the blood–brain barrier^25^ and, in this context, some trials are evaluating abemaciclib in ER-positive BC patients with brain metastases. Preclinical and early-phase trials highlighted the activity of abemaciclib in HR-positive BC, supplying a rationale for further development.^24,26^

The Phase II open-label MONARCH-1 trial evaluated abemaciclib monotherapy in endocrine-resistant, chemotherapy-pretreated, postmenopausal mBC patients, showing objective response rate, clinical benefit rate, median PFS, and median OS of 19.7%, 42.4%, 6.0, and 17.7 months, respectively.^27^ Given that results, abemaciclib has been also investigated in combination with endocrine therapy. In the randomized Phase III MONARCH-2 trial, the combination of abemaciclib and fulvestrant significantly prolonged median PFS as compared with fulvestrant plus placebo in endocrine-resistant, chemotherapy-naive, pre- and postmenopausal mBC patients (16.4 versus 9.3 months).^28^ To date, no data regarding OS of treated patients have been published. Subgroup analyses showed no statistically significant differences in PFS benefit between younger and older patients. Furthermore, abemaciclib was also tested in endocrine-sensitive, postmenopausal mBC patients. The randomized Phase III MONARCH-3 trial tested abemaciclib plus anastrazole/letrozole versus anastrazole/letrozole plus placebo, reporting an improved median PFS in abemaciclib group compared with placebo group (28.1 versus 14.7 months).^29,30^ Subgroup analysis showed no differences in efficacy between younger and older patients. OS data were still immature at time of primary analysis.

## Ribociclib: MONALEESA trials

Ribociclib is another highly-selective, reversible small-molecule inhibitor of CDK4/6. Ribociclib showed remarkable preclinical efficacy as well as acceptable safety profile and preliminary signs of clinical activity in variety of solid tumors, including HR-positive BC.^31–33^ Therefore, several trials investigated ribociclib in ER+/HER2- mBC.

In the Phase III MONALEESA-2 trial, endocrine-sensitive, treatment-naive for metastatic disease, postmenopausal mBC patients were randomized to ribociclib plus letrozole versus letrozole plus placebo. Median PFS was significantly longer in ribociclib arm than in placebo arm (25.3 versus 16.0 months).^34^ Furthermore, ribociclib has also been tested in endocrine-resistant, postmenopausal mBC patients. The randomized Phase III MONALEESA-3 trial testing ribociclib plus fulvestrant versus fulvestrant plus placebo reported an improved median PFS in the ribociclib arm (20.5 vs 12.8 months).^35^

At last, ribociclib was also evaluated in the first-line setting of pre- and perimenopausal mBC patients. In the Phase III MONALEESA-7 trial, ribociclib plus anastrazole/letrozole and LH–RH analog significantly increased median PFS compared with anastrazole/letrozole and LH–RH analog plus placebo (23.8 vs 13.0 months).^36^ Recently, the updated analysis of this study showed that the addition of ribociclib to endocrine therapy significantly prolonged OS as compared with endocrine therapy alone with an estimated OS at 42 months of 70.2% in the ribociclib group and 46.0% in the placebo group (hazard ratio for death, 0.71; log-rank *P* = 0.00973).^37^ To date, MONALEESA-7 has been the first trial investigating CDK4/6 inhibition in mBC to demonstrate an OS benefit. In addition, this trial enrolled the larger cohort of premenopausal patients, confirming the results previously obtained mainly in the postmenopausal population.

## How to select the CDK 4/6 inhibitor in clinical practice?

### Pharmacological differences

Despite comparable results in terms of clinical efficacy, the three CDK4/6-Is present substantial pharmacological differences, as summarized in Table 2.

**Table 2:** Pharmacological characteristics of CDK4/6 inhibitors

	Palbociclib (pd-0332991; ibrance, pfizer)	Abemaciclib (ly2835219; verzenio, lilly)	Ribociclib (lee011; kisquali, novartis)
Chemical structure			
Ic50 (nm)			
Cdk4-cyclin d1	11	2	10
Cdk6-cyclin d1-2-3	15	10	39
Absorption	Increased with high-fat, high-calorie food	NR	NR
Distribution	2583 L	690.3 L	1090 L
Metabolism	Liver (cyp3a and sult2a1)	Liver (cyp3a4)	Liver (cyp3a4)
Excretion	Feces (~74%)	Feces (~81%)	Feces (~69%)
	Urine (~18%)	Urine (~3%)	Urine (~23%)
Bioavailability	46%	45%	NR
Time to peak (hours)	6–12	8	1–4
Half-life elimination (hours)	29 ± 5	18.3	30–55
Protein binding	~85%	93–98%	~70%
Mtd/rp2d	125/125 mg/day on a 21-of-28-day schedule	200 mg twice daily	900/600 mg/day on a 21-of-28-day schedule
Dlts	Neutropenia	Fatigue	Neutropenia, asymptomatic thrombocytopenia, mucositis, pulmonary embolism, hyponatremia, QTcF, prolongation (> 500 ms), increased creatinine
Route of administration	Oral	Oral	Oral
Recommended dose	125 mg once daily for 21 days, followed by 7 days off, repeat every 28 days	150 mg twice daily	600 mg once daily for 21 days, followed by 7 days off, repeat every 28 days
		Dose modifications	
Renal impairment			
Crcl > 15 ml/min	No dosage adjustament	No dosage adjustament	No dosage adjustament
Crcl ≤ 15 ml/min	NR	NR	NR
Esrd	NR	NR	NR
Hepatic impairment*			
Mild/moderate	No dosage adjustament	No dosage adjustament	No dosage adjustament
Severe	Reduce dose to 75 mg	Reduce dose to once daily	Reduce dose to 400 mg

Chemical structures are available online at: https://pubchem.ncbi.nlm.nih.gov. Data about pharmacological characteristics of the three drugs are available online at: https://www.drugs.com

*DLT* dose-limiting toxicity, *ESRD* end-stage renal disease, *IC50* half maximal inhibitory concentration, *MTD* maximum tolerated dose, *NR* not reported, *RP2D* recommended phase II dose

*Mild, moderate, and severe hepatic impairment refers to Child-Pugh classes A, B, and C, respectively

Palbociclib, ribociclib, and abemaciclib are orally administered small molecules, which inhibit CDK4 and CDK6 by binding to the ATP clefts of these molecules. Palbociclib and ribociclib have similar potencies in terms of CDK4 and CDK6 inhibition with reported IC50s of 11 and 15 nm (for palbociclib) and 10 and 39 nm (for ribociclib), respectively. On the other hand, abemaciclib studies report high IC50s: 2 nm for CDK4 and 10 nm for CDK6. Concerning target activity, palbociclib and ribociclib are able to inhibit only CDK4 and CDK6, whereas abemaciclib has an additional activity against CDK9.^3^ CDK9 is an enzyme implicated in the regulation of a broad spectrum of transcriptional events as well as in embryogenesis and cell proliferation process.^38^ This activity against CDK9 could in part explain the clinical efficacy of abemaciclib monotherapy showed in MONARCH-1 trial^27^ and the specific gastrointestinal toxicity that is less pronounced with ribociclib and palbociclib.^39^ In contrast, ribociclib and palbociclib have greater lipophilicity and different binding-site side chains compared with abemaciclib. These features could partially clarify the reduced number of off-target interactions for palbociclib and ribociclib.^40^

Concerning drug administration, palbociclib and ribociclib are administered for 21 days, followed by 7 days off treatment, at the standard doses of 125 mg once daily and 600 mg once daily, respectively.^41,42^ Conversely, abemaciclib dosing is 200 mg every 12 hours, given continuously.^43^

All three drugs are mainly metabolized at liver, mainly through oxidation by the cytochrome P450 3A4 (CYP3A4). In addition, palbociclib undergoes hepatic metabolism involving also the sulfotransferase enzyme SULT2A1. Given that pharmacological property, the concomitant use of strong CYP3A inhibitors (e.g., clarithromycin, itraconazole, ketoconazole, ritonavir, and grapefruit juice) should be avoided. In patients who need simultaneously administration of a strong CYP3A inhibitor, a dose reduction of palbociclib to 75 mg is recommended. Moreover, also the administration of strong CYP3A inducers (e.g., rifampin, phenobarbital, St. John’s Wort) should be avoided in order to prevent palbociclib plasma levels decreasing. As ribociclib and abemaciclib are metabolized through oxidation via CYP3A4, the same recommendations reported for palbociclib remain valid.^42,43^ Interestingly, a common clinical scenario could be represented by patients who need anticoagulant treatment. As reported in recent analysis, patients receiving CDK4/6-I have an augmented risk of thromboembolic events,^44^ ranged from 0.6 to 5% in the evaluated trials. Given the increasing use of direct oral anticoagulants (DOACs) in cancer patients and considering that these drugs have a liver metabolism,^45,46^ the concomitant administration of CDK4/6-I and DOAC has to be carefully discussed in absence of data about potential pharmacological interactions. Further data on this relevant topic are strongly warranted.

In cases of mild hepatic impairment (Child-Pugh A and B), no dose adjustments are required. However, in patients with severe hepatic dysfunction (Child-Pugh C) it is recommended to start with reduced doses for all CDK4/6-Is. At last, pharmacokinetic data about all three drugs in patients affected by severe renal impairment (CrCl <30 ml/min) are still not available. In this context, further studies evaluating safety and clinical efficacy of CDK4/6-Is in this patient subgroup are strongly encouraged. Of interest, an increase in serum creatinine has been associated with abemaciclib administration.^27^ This effect is related to an on-target effect of abemaciclib, which competitively inhibits the renal tubular secretion transporters OCT2 and MATE, without affecting glomerular filtration.^47^

### Toxicities

**Fig. 2:**
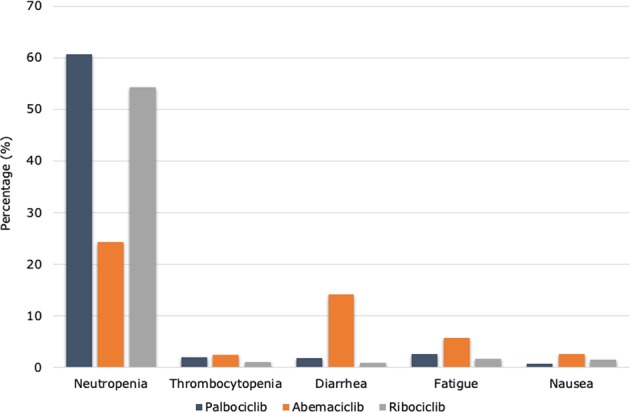
Common grade 3–4 adverse events reported in pivotal trials of CDK4/6 inhibitors

**Fig. 3:**
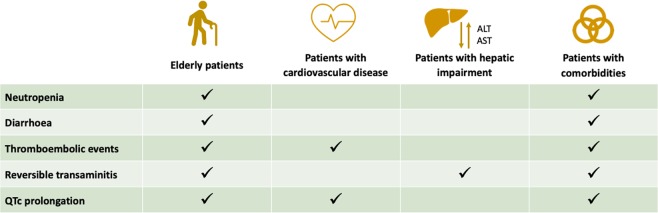
Effect of toxicity in selecting CDK4/6 inhibitors

In terms of toxicity profile, the three CDK4/6-Is present few, but consistent differences. Overall, palbociclib and ribociclib presents with a predominant bone marrow toxicity. Conversely, abemaciclib administration has been correlated with gastrointestinal symptoms and less-pronounced hematologic toxicity (Table 3 and Figs 2 and 3).

**Table 3:** Common toxicities of CDK 4/6 inhibitors reported in pivotal trials

		Neutropenia	Febrile neutropenia	Leukopenia	Fatigue	Anemia	Thrombocytopenia	Arthralgia	Diarrhea	Nausea	Vomiting
All grades	Paloma-1	74	NR	43	40	35	16	23	21	25	14
Grades 3/4		54		19	4	6	2	1	4	2	0
All grades	Paloma-2	79.5	1.8	39	37.4	24.1	15.5	33.3	26.1	35.1	15.5
Grades 3/4		66.5		24.8	1.8	5.4	1.6	0.7	1.4	0.2	0.5
All grades	Paloma-3	78.8	1	45.5	38.0	26.1	19.4	13	19.1	29.0	14.5
Grades 3/4		62.0		25.2	2.0	2.6	2.3	0.3	0	0	0.3
All grades	Monarch-1	87.7	0.7	90.9	65.2	68.5	41.1	NR	90.2	64.4	34.8
Grades 3/4		26.9		27.7	12.9	0	2.3	NR	19.7	4.5	1.5
All grades	Monarch-2	46.0	0.9	28.3	39.9	29.9	15.6	11.6	86.4	45.1	25.9
Grades 3/4		26.5		8.8	2.7	7.2	3.4	0.2	13.4	2.7	0.9
All grades	Monarch-3	41.3	0.3	20.8	40.1	28.4	36.2	NR	81.3	38.5	28.4
Grades 3/4		21.1		7.6	1.8	5.8	1.9	NR	9.5	0.9	1.2
All grades	Monaleesa-2	74.3	1.5	32.0	36.5	18.6	NR	27.2	35.0	51.5	29.3
Grades 3/4		59.3		21	2.4	1.2	NR	0.9	1.2	2.4	3.6
All grades	Monaleesa-3	69.6	1.0	28.4	31.5	17.2	NR	24.0	29.0	45.3	26.7
Grades 3/4		53.4		14.1	1.7	3.1	NR	0.6	0.6	1.4	1.4
All grades	Monaleesa-7	76	2	31	23	21	6	30	20	32	19
Grades 3/4		61		14	1	3	1	1	1	1	1

In PALOMA trials, the most commonly reported grade 3–4 adverse events (AEs) were neutropenia (54–66%), leukopenia (19–25%), anemia (2–6%), and fatigue (2–4%). As for palbociclib, the hematological AEs are common with ribociclib. Differently, the MONALEESA trials highlighted other relevant toxicities. Grade 3–4 aspartate aminotransferase and alanine aminotransferase elevations occurred in 5–10% of patients treated with ribociclib. Regarding liver abnormalities, liver function tests should be obtained before initiating treatment with ribociclib and monitored at the beginning of each subsequent cycle. Furthermore, ribociclib administration has been associated with QT interval prolongation in ~1–3% of treated patients.^34–36^ In this context, the use of ribociclib in patients with significant risk of developing QTc prolongation, such as those affected by long QT syndrome, uncontrolled or significant cardiac disease, recent myocardial infarction, congestive heart failure, unstable angina, bradyarrhythmias, and uncontrolled electrolyte abnormalities, should not receive ribociclib. In addition, the avoidance of QT-prolonging agents, including fluoroquinolones, ketoconazole, itrokonazole, some antidepressant (citalopram, escitalopram, mirtazapine, venlafaxine) and some antipsychotic agents (haloperidol, sulpiride, pimozide, chlorpromazine),^48^ as well as an adequate supplementation for electrolyte alterations are recommended.^49^ Electrocardiogram has to be performed at baseline and repeated at day 14 of the first cycle, at the beginning of the second cycle, and then as clinically indicated; electrolytes have to be dosed at baseline and monitored during the treatment in all patients.

Despite the high frequency of neutropenia for all CDK4/6-Is (in particular, palbociclib and ribociclib), febrile neutropenia and other serious infections have been rarely reported in clinical trials, ranged from 1 to 2% in palbociclib and ribociclib trials and <1% in abemaciclib trials (Table 1). Dose reductions for palbociclib and for persistent neutropenia are common. Reassuringly, dose modifications for grade 3–4 neutropenia have not a deleterious effect on PFS.^50^

At last, abemaciclib had lower rates of neutropenia but greater degrees of diarrhea compared with the other agents. However, the majority of patients did not require treatment modification for this kind of toxicity, being well managed with antidiarrhoeic medications.

### Patient-reported outcomes

As shown above, the addition of CDK4/6-I to endocrine therapy significantly contributes to improve response rates and survival times in ER+/HER2- mBC patients. However, it is critical to understand the impact of these therapies on QoL of treated patients. As such, patient-reported outcomes (PROs) has been demonstrated to be integral components for tabulating symptomatic toxicities, enabling symptoms that could be underestimated by clinicians, and assessing benefit–risk of new treatment regimens.^51^ Some trials testing CDK4/6-Is reported PROs together with results on efficacy and toxicity.

In the PALOMA-2 trial, the addition of palbociclib to letrozole maintained health-related QOL, evaluated by means Functional Assessment of Cancer Therapy (FACT)-Breast Total, FACT-General Total, and EuroQOL-5 dimensions scores.^52^ In addition, the palbociclib group presented an improvement in pain scores. In both arms, patients without signs of clinical or radiological disease progression presented with a delayed worsening of FACT-Breast Total score compared with those with progression. Comparable results were obtained in the PALOMA-3 trial: overall global QoL scores, evaluated through the European Organization for Research and Treatment of Cancer Quality-of-Life C30 (EORTC QLQ-C30) questionnaire, significantly favored the palbociclib plus fulvestrant group along with a greater improvement from baseline in pain control.^53^

As for palbociclib trials, PROs were also reported for ribociclib studies. In the MONALEESA-2 trial, on-treatment HRQoL scores, using the EORTC QLQ-C30 questionnaire, were consistently maintained from baseline and were similar between the two arms.^54^ A clinically meaningful reduction in pain score was observed in the ribociclib arm. Similar results were reported in the MONALEESA-3 trial whereby the combination of ribociclib and fulvestrant maintains QoL compared with fulvestrant plus placebo.^55^ Moreover, in the MONALEESA-7 trials median time to definitive deterioration of QoL, measured by the EORTC QLQ-C30, was not reached in the ribociclib group compared with 21.2 months in the placebo group (HR 0.70, *p* = 0.004). In addition, a clinically meaningful improvement from baseline in EORTC QLQ-C30 pain score was observed in the ribociclib group.^36^

Regarding abemaciclib trials, PROs are available only for the MONARCH-2 study. In this latter, no significant differences in HRQoL, using the EORTC QLQ-C30, BR-23, and Brief Pain Inventory short form, were observed between the two arms.^56^ However, diarrhea, appetite loss, nausea, and vomiting were worse in the abemaciclib group.

## Discussion

The relevant results obtained with CDK4/6-Is in ER-positive mBC led to the approval of all three agents by the regulatory agencies. At present, several open issues remain about the proper application of these drugs in daily medical practice.

As previously showed, the administration of CDK4/6-Is, mainly in combination with endocrine therapy, produced substantial benefit in terms of PFS both in endocrine-sensitive (PALOMA-2, MONARCH-3, MONALEESA-2, and MONALEESA-7 trials) and in the endocrine-resistant (PALOMA-3, MONARCH-2, and MONALEESA-3 trials) mBC patients. Of note, MONALEESA-7 has been the first trial, which evaluated CDK4/6-Is in mBC, to demonstrate a significant improvement in terms of OS.^37^ Considering exclusively the trials testing the addiction of CDK4/6 inhibition in endocrine-resistant patients, the PALOMA-3 study results highlighted that patients with previous sensitivity to endocrine therapy presented had an OS benefit of about 10 months with addiction of palbociclib, although this improvement was not seen in the overall population.^21^ In this way, further OS data from clinical trials testing CDK4/6-Is in ER-positive mBC are strongly awaited.

Concerning the safety profile of CDK4/6-Is, some differences have been reported and have to be considered at the time of drug choice. Abemaciclib is associated with less-hematologic toxicity and more gastrointestinal symptoms, whereas palbociclib presents a high percentage of grade ¾ neutropenia. In addition, ribociclib has a potential for QT interval prolongation. In clinical practice, the different safety profiles have to be carefully evaluated by physicians prior to prescribe one of these drugs. For example, in patients with cardiac diseases, ribociclib could not be the right therapeutic choice as well as abemaciclib might not be the right drug for subjects with preexistent gastrointestinal comorbidities. Regarding PROs, CDK4/6-I use in first-line setting has not been associated with an improvement in HRQoL,^52,54^ except in MONALEESA-7 where median time to definitive deterioration was significantly reduced in ribociclib arm.^36^ Interestingly, the PROs evaluation in PALOMA-2 indicates that disease progression was associated with degradation of HRQoL, regardless of palbociclib treatment.^52^ In the second-line setting, HRQoL improvement has also been observed.^53^ Noteworthy, all trials reported a better pain management in patients treated with the combination of CDK4/6-I and endocrine therapy.

Another relevant point concerns the implementation of tissue and/or serum biomarkers, which could adequately select patients candidate to receive combinatorial therapeutic strategies (CDK4/6-I + endocrine agent ± other agent) and those who can be can be treated by endocrine therapy alone. In this regard, the recent finding of high CCNE1 expression as resistance mechanism to palbociclib^23^ opens the door to further prospective clinical trials investigating CDK4/6 inhibition in biomarker-defined mBC populations. Furthermore, the optimal therapeutic sequence as well as the right treatment after progression on CDK-I remain important open questions. Ongoing clinical trials, including PARSIFAL (NCT02491983) and SONIA (NCT03425838), will provide novel and substantial insights in these clinical contexts.

The substantial results obtained with CDK4/6 inhibition in the advanced setting prompted clinical research to implement these agents also in early BC. To date, several clinical trials are testing the addition of CDK4/6-Is in neoadjuvant and adjuvant settings of early BC patients, mainly in combination with endocrine therapy. However, several issues have to be considered about the role of neoadjuvant treatment of ER-positive BC. The majority of the neoadjuvant trials in BC are designed with the pathological complete response (pCR) as primary end-point. Although the pCR is a recognized surrogate marker of long-term survival in HER2+ and triple-negative BC,^57^ its value in ER-positive disease is controversial. Considering the low rates of pCR after endocrine neoadjuvant therapy^58^ as well as the possibility to achieve good survival outcomes with standard (or extended) adjuvant endocrine therapy and to tailor the adjuvant treatment on the basis of several validated prognostic gene signatures,^59^ other markers of biological and clinical activity have been implemented and evaluated in different clinical trials testing neoadjuvant therapies in ER-positive disease. These include the changes in cell proliferation rate evaluated by means the Ki67 index, the preoperative endocrine prognostic index—a composite score of post-treatment ER, Ki67, tumor size, and axillary nodal status, and the residual cancer burden index.^59^ Several trials, such as NeoPalAna,^60^ neoMONARCH,^61^ N007,^62^ and NeoPAL,^63^ showed that the addition of CDK4/6-Is led to cell cycle arrest, defined as a Ki67 proliferation index <2.7%, 2 weeks after the beginning of treatment in 68–87% of patients as compared with 14–26% of patients treated with endocrine therapy alone.^64^ In another presurgical, window-of-opportunity study, ribociclib also demonstrated a remarkable biological activity in ER-positive/HER2-negative early BC patients, with a significant reduction in proliferation index and CDKs-Rb-E2F pathway proteins.^65^ However, as in other trials that tested neoadjuvant endocrine therapy in BC, these studies did not show substantial improvements in pCR rates with the addition of CDK4/6-Is. Moreover, the limited information about the association between Ki67 level changes and long-term survival outcomes as well as the possible increase in Ki67 value when CDK4/6 inhibition was stopped in the preoperative period represent relevant issues to clarify in further studies.

Besides HR-positive disease, CDK4/6-Is are currently investigating in HER2-overexpressing and triple-negative BCs.^47^ Furthermore, the recently reported immunomodulatory activity of the CDK4/6-Is in preclinical tumor models^66^ paved the way to test these agents in combination with immune checkpoint inhibitors. Several trials are currently testing these therapeutic strategies in solid tumors, including BC. At last, the profound cross-talk between CDK4/6 and the PI3K-AKT-mTOR pathway provided a rationale for implementing combinatorial therapeutic strategies.^67^

In conclusion, palbocilib, ribociclib, and abemaciclib obtained relevant results and are actually the standard of care for the treatment of HR-positive mBC patients in first, second, and beyond lines of therapy.^11^ Because comparable results in terms of clinical efficacy as well as substantial differences in side effects have been provided, clinical choice of one of these drugs should rely on patient preference, administration schedules, and concomitant diseases. Ongoing clinical trials will define the role of CDK4/6 inhibition in early BC as well as in other BC subtypes, including HER2-positive and triple-negative diseases. Given the similar results in clinical trials testing CDK4/6-Is, observational real-world studies will be able to provide new insights for the implementation of these drugs in the clinical practice.

## Data Availability

Source data for all figures and tables are provided in the paper. No new data sets have been generated or analyzed for this article.
